# Multiple thromboembolism with multiple causes in a 69-year-old woman: a case report

**DOI:** 10.1186/1752-1947-5-186

**Published:** 2011-05-14

**Authors:** Luigi Iuliano, Maria Misuraca, Alessandro Varroni, Massimo Raponi, Marco Massucci, Alessandro Pagnanelli, Giuseppe Cimino, Giovanni Bertoletti

**Affiliations:** 1 Department of Medical Sciences and Biotechnology, Vascular Medicine and Atherothrombosis Laboratory, Sapienza University of Rome, corso della Repubblica 79, IT-04100 Latina, Italy; 2 Unit of Vascular Medicine, Goretti Hospital, via Guido Reni 3, IT-04100 Latina, Italy; 3 Unit of Vascular Surgery, Goretti Hospital, via Guido Reni 3, IT-04100 Latina, Italy; 4 Unit of Cardiology, Goretti Hospital, via Guido Reni 3, IT-04100 Latina, Italy; 5 Department of Cellular Biotechnology and Hematology, Sapienza University of Rome, via Benevento 6, IT-00161 Rome, Italy

## Abstract

**Introduction:**

Aggressive, recurrent embolisms require accurate etiologic diagnosis. We describe the case of a 69-year-old Italian Caucasian woman with recurrent arterial embolisms in whom several sources and triggers of thrombosis were detected.

**Case presentation:**

The patient, a 69-year-old Italian Caucasian woman, presented with a systemic embolism that was initially attributed to atrial fibrillation. The recurrence of embolisms despite anti-thrombotic therapy prompted a re-evaluation of the clinical presentation. New potential causes of thrombosis emerged in this patient, including thrombocytosis associated with the *JAK2 V617F *mutation and the very rare mural thrombosis of the descending aorta. A mural thrombus in the pulmonary artery was detected contiguous with the aortic mural thrombosis, raising the possibility of a clinically silent ductus Botalli as the initiating event. The patient was treated with warfarin, aspirin, hydroxyurea, and surgery.

**Conclusions:**

The diagnosis was achieved via systematic use of imaging procedures and reconsideration of blood tests performed to explore the diagnosis of thrombosis. This allowed a deeper and more detailed analysis of the case beyond the conventional approach, which would have aimed to identify one cause for the condition at hand, in this case, atrial fibrillation. The broader approach that we used resulted in the diagnosis of multiple embolisms from multiple sites and multiple causes.

## Introduction

The etiologic diagnosis of arterial embolisms is challenging. Embolisms may arise from cardiac thrombi, patent foramen ovale, atrial septal aneurysms associated with paradoxical embolisms, and complicated atherosclerotic lesions of the aortic arch. The recurrence of embolisms while the patient is undergoing an anti-thrombotic regimen may underline multiple causes of thrombosis, including exceptional cases of mobile thrombi in the aortic arch in the absence of diffuse atherosclerosis that have been reported recently [1]. Transesophageal echocardiography is of valuable help for the detection of mobile thrombi with high embolic potential. We describe the case of a 69-year-old Italian Caucasian patient with recurrent arterial embolisms in whom several sources and triggers of thrombosis were detected.

## Case presentation

A 69-year-old Italian Caucasian woman arrived at the emergency department presenting with sudden onset of lower left limb pain. The patient reported no dyspnea, chest pain, weight loss, nausea, abdominal pain, hematuria, myalgias, or arthralgias. Her medical history was notable for type 2 diabetes mellitus and chronic atrial fibrillation. She had no history of coronary heart disease, hypertension, or dyslipidemia, and she did not smoke. Her medications included digoxin and repaglinide, but not anti-thrombotic drugs.

The patient's physical examination revealed that she appeared to be in good condition, but she reported discomfort due to pain in the left leg. The patient's blood pressure was 125/85 mmHg, and her heart rate was 80 beats/minute and irregularly irregular. Her respiratory rate was 22 breaths/minute, her oxygen saturation was 98% while the patient was breathing ambient air, and her body temperature was 36°C.

In her left leg, femoral, popliteal, and tibial pulses were absent, with pale and cool feet and with slow capillary return. Her right leg was absent of tibial pulse. Her Ankle Brachial Pressure Index was 0 on the left and 0.4 on the right. The motor and sensory functions of the legs were preserved.

A transesophageal echocardiogram revealed enlargement of the left atrium with a thrombus in its appendange and enlargement of the right atrium. She had no signs of atrial shunt or detectable atherosclerosis in the proximal aorta. A diagnosis of embolizing non-valvular atrial fibrillation was confirmed, and the patient underwent lower left limb Fogarty thromboembolectomy, that successfully restored distal blood flow. The patient was discharged after two days to a low molecular weight heparin regimen as bridging therapy for a warfarin regimen.

After one week, the patient returned to the emergency department with acute ischemic symptoms in her left leg. Angiography was performed, which showed an apparently normal wall of the abdominal aorta, right iliac and femoral artery branches, obstruction of the left external iliac artery and total obstruction of the left femoral artery branches, and obstruction of the right popliteal artery and of the tibial-peroneal axes on both sides. Urgent embolectomy was again performed with subsequent restoration of blood flow, and the patient was discharged under warfarin therapy.

After four weeks, the patient returned with left leg pain at rest. A search for a hypercoagulable state revealed normal thrombin, pro-thrombin, and partial thromboplastin times; normal anti-thrombin III, protein S, and protein C antigens; and a normal protein C pathway, activated protein C resistance, and homocysteine level. Screening for anti-cardiolipin antibodies and lupus anti-coagulant were negative, and her anti-nuclear antibody test was negative. Her erythrocyte sedimentation rate was 8 mm/hour. Her blood cell count revealed normal amounts of red and white cells, and her platelet count was 850,000/μl.

**Figure 1:**
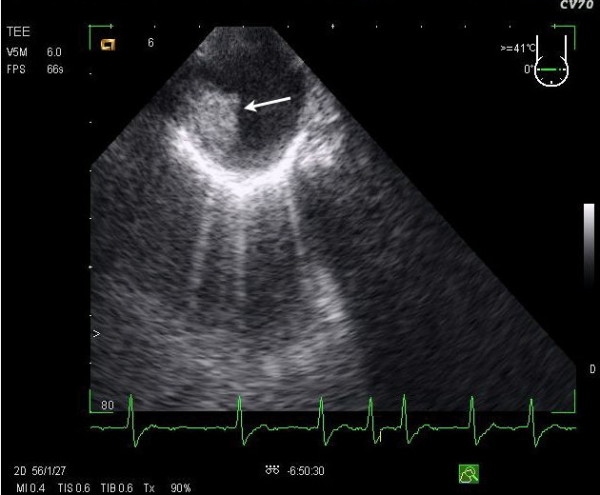
**A trans-esophageal echocardiographic scan showing an intra-luminal pyramidal lesion 1.4 cm×1.5 cm in size with an irregular surface on the anterior wall of the descending aorta (arrow)**.

**Figure 2:**
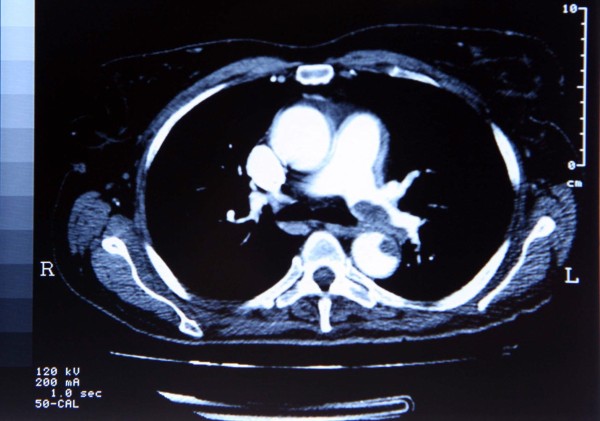
**A computed tomography slice of the thorax showing a mural aortic thrombus in the anterior wall of the descending aorta and a mural thrombus in the left pulmonary artery localized in close proximity to the aortic thrombus**.

Transesophageal echocardiography showed resolution of the atrial thrombus; however, an intra-luminal pyramidal lesion (1.4 cm×1.5 cm in size) with an irregular surface was seen on the anterior wall of the descending aorta (Figure 1). Computed tomography (Figure 2) with contrast medium confirmed the presence of a mural aortic thrombus in the anterior wall of the descending aorta extending 1.5 cm distally, a mural thrombus in the left pulmonary artery localized in close proximity to the aortic thrombus, a lung infarction in the inferior left lobe, an infarction in the spleen, sparse atherosclerotic plaques in the abdominal aorta and the right femoral artery, occlusion of the left external iliac artery, and occlusion of the right internal iliac artery. There were no signs of venous thrombosis.

The patient underwent surgical treatment consisting of an endovascular exclusion of thrombus with a Zenith 32 mm×80 mm endoprosthesis (Cook, Inc., Bloomington, IN) implanted just after the emergence of the left anonym subclavian artery, a subsequent thromboembolectomy of the left external iliac artery, and a femoro-popliteal bypass. The patient was discharged after a few days under a low molecular weight heparin regimen as bridging therapy for a warfarin regimen, as well as aspirin. Additional therapy included β-blockers and hydroxyurea, an anti-neoplastic drug acting on nucleic acid synthesis to reduce her platelet count. Her platelet count decreased to 300,000/μl.

Two months later an echocardiographic Doppler study revealed the patency of the bypass and the left femoral axis, which previously had been treated by embolectomy. Two months later the patient presented with left leg pain while she was being treated with warfarin. An echocardiographic Doppler scan showed obstruction of the popliteal bypass. Therapy with aspirin, clopidogrel, and warfarin was initiated. Out-patient follow-up was arranged, and eight months later the patient did not report any further embolization.

## Discussion

Our patient presented with recurrent overt episodes of peripheral embolization and subclinical pulmonary and spleen embolism. The first reported episode of peripheral embolism in our patient was associated with left atrial appendange thrombosis as a consequence of atrial fibrillation that was not anti-thrombotic prophylaxis. Atrial fibrillation is the most prevalent cardiac dysrhythmia and is a strong independent risk factor for both systemic embolism and stroke [2]. Systemic embolic events associated with atrial fibrillation are cerebral in 85% of cases and peripheral in 15% [3]. According to the current guidelines [4], the patient presented here should have been treated with aspirin or warfarin.

In our patient, the subsequent embolization occurred after we initiated anti-coagulation therapy. This therapy was associated with the resolution of atrial thrombosis and the appearance of a mural thrombus localized in the descending aorta as a consequence of the underlying thrombocytosis and, eventually, some degree of atherosclerosis. Peripheral embolization is of cardiac origin or due to atherosclerosis of the abdominal aorta in 85% of cases [5]. Peripheral embolisms associated with atherosclerosis of the abdominal aorta generally occur in the presence of advanced and complicated lesions. The angiogram in our case, however, did not show appreciable atherosclerosis of the abdominal aorta.

The initiation of anti-coagulation therapy is not sufficient to fully prevent further episodes of embolization from the atrial thrombus. Patients with atrial fibrillation and dense spontaneous echocontrast have a high likelihood of cerebral embolism (22%) or death, despite the use of oral anti-coagulation therapy [6]. In the first days after initiation of anti-coagulation treatment, the risk of embolization remains high. A transient pro-thrombotic state can be induced by excessive doses of warfarin, which rapidly depletes circulating protein C [7]. On the other hand, embolectomy is associated with a recurrence rate of thrombosis of 4% to 7% [8].

Recurrent thromboembolism under warfarin treatment should prompt the clinician to search for other factors, including a hypercoagulable state or vasculitis. In addition, trans-esophageal echocardiography should be repeated to assess the state of the previously recognized atrial thrombosis. The only relevant alteration, in the blood analysis results in our patient, was the marked increase in the number of platelets, which was not present at the first admission.Thrombocytosis is *per se *complicated by major thrombosis, with a rate as high as approximately 50% [9]. Two possible explanations could account for our patient's normal platelet count at admission: either this was a reactive thrombocytosis or thrombocytosis was misdiagnosed at admission for the eventual occurrence of artefactual platelet count [10]. In the event of preexisting thrombocytosis, we tested the patient for positivity for the *JAK2 V617F *mutation to establish whether it was essential thrombocytosis according to the recently revised diagnostic criteria [11]. Essential thrombocytosis, which is one of the conditions of myelo-proliferative disorders, is associated with *JAK2 *mutation in up to 40% to 50% of cases. Actually, a polymerase chain reaction assay confirmed the presence of the *JAK2 V617F *mutation. JAK2 is a member of the Janus kinase family that is implicated in cellular signaling in connection with several receptors. The change of valine to phenylalanine at the 617 position is a gain of function mutation that renders hematopoietic cells more sensitive to growth factors such as thrombopoietin.

The imaging results in the present case revealed the disappearance of atrial thrombosis and the appearance of new thrombi attached to the wall of the descending aorta and in the pulmonary artery. The thrombus in the descending aorta, also known as the mural thrombus, is a very rare cause of systemic embolization. A mural thrombus in the pulmonary artery has never been reported. The atrial thrombus and the mural thrombi in the descending aorta and in the pulmonary artery can be associated with limb, spleen, and lung embolization. Fortunately, the patient's brain circulation was saved from embolic events, as the aortic mural thrombi grew after the emergence of the left subclavian artery. Eventually, the atrial emboli were too large in diameter to enter the carotid branch and traveled downstream. Thrombocytosis and the eventual alteration of the arterial wall favored the diagnosis of aggressive thromboembolism in this patient.

Thrombosis of the aortic arch is an infrequent cause of systemic embolisms. Machleder *et al*. [12] reported 10,671 consecutive autopsies in which aortic mural thrombus was identified in 48 of the cases (a 0.45% incidence) in aortas of normal caliber and configuration. Thirty-eight patients had mural thrombi confined to the abdominal aorta, one had a mural thrombus in the thoracic aorta, and nine had mural thrombi in both the thoracic and the abdominal aorta [12]. In 1997, Laperche *et al*. [1] published a large series of patients ranging in age from 26 to 61 years with unexplained arterial embolisms with mobile aortic arch thromboses detected by trans-esophageal echocardiography. Of the 27,855 examinations, 23 cases were detected (a 0.08% incidence) [1]. In their study, a large proportion of cases was associated with a certain degree of atherosclerosis, but one patient did not have measurable atherosclerosis, and 90% of cases were associated with atherosclerosis risk factors. In this series of cases, intravenous unfractionated heparin was used to manage embolic events; however, in 17% of the cases, embolisms recurred despite heparin administration. Forty-three percent of the patients had multiple embolisms affecting more than one territory.

Several authors have reported the association of aortic mural thrombi with hypercoagulable states, including resistance to protein C and hyperhomocysteinemia [13]. However, it is likely that most, if not all, aortic mural thrombi occur because of an endothelial perturbation triggered by some grade of atherosclerosis or inflammation, albeit undetectable using current imaging procedures.

Accordingly, most patients diagnosed with aortic mural thrombi are positive for atherosclerosis risk factors, including our patient, who has type 2 diabetes mellitus. Taken together, these elements satisfactorily explain the occurrence of aortic mural thrombus in our patient. In this case, however, the occurrence of aortic mural thrombus along with contiguous pulmonary artery thrombosis is novel and has never been reported in the literature. Thrombosis in the pulmonary arteries was not caused by venous thromboembolism, which was excluded by computed tomographic angiography. We have presented the case of a patient with a mural thrombus in the pulmonary artery, which was likely triggered by alteration of the endothelial lining in a manner similar to the mural thrombus of the aorta. Atherosclerosis can be excluded as the underlying disease, as pulmonary arteries are not typically affected by this disease. The question, then, is why our patient developed mural thrombosis of the pulmonary artery. The spatial and temporal contiguity of the two mural thromboses, in the aorta and the pulmonary artery, may help in addressing this question.

The computed tomographic image in Figure 1 shows that the aortic and pulmonary artery walls involved in mural thrombosis are in close contiguity with one another. In addition, it is likely on the basis of the clinical evolution that the two thromboses developed almost simultaneously. Two possible causes that may act trans-arterially are inflammatory or infectious processes. This hypothesis, however, may not be accurate, as we did not find any marker of inflammation or infection in the blood, such as increases in white blood cell count, C-reactive protein, anti-nuclear antibody titers, erythrocyte sedimentation rate, or body temperature.

One possible explanation involve the mechanical stress at the point of contact between the two vessels, with a point of tension during the systolic wave, which eventually increases from the potential arterial stiffness in a patient with multiple risk factors for atherosclerosis. Mechanical stress of the wall could affect the endothelial homeostasis, with a shift toward thrombogenesis.

It should be emphasized that thrombosis occurred at an interface of the two vessels in the area of the ancestral communication between the systemic and pulmonary circulation: the ductus Botalli. Although its distribution and size may vary, the ductus Botalli normally extends antero-superiorly from the anterior margin of the descending aorta to the superior margin of the pulmonary artery adjacent to the orifice of the left pulmonary artery, and obliquely from right to left [14]. Patent ductus Botalli (PDB) in young patients, an isolated anomaly and the most common lesion in the field of congenital cardiac diseases, is readily recognized and can be surgically corrected with minimal risk. Occasionally, the ductus Botalli escapes detection, and the patient survives to adulthood. In a series of cases of PDB in patients over 50 years old at the Mayo Clinic from 1945 to 1983, two-thirds of the patients with PDB were women [15]. Some cases of PDB have been reported in adults over 80 years of age, including a case of a 91-year-old woman in Japan who survived without ductus closure [16].

A small PDB causes no symptoms, and a person with a small defect has a normal life expectancy [17]. However, small PDB is associated with an elevated risk of infectious endocarditis, and thromboembolic events are the most serious reported complications [18].

A communication, even small and not affecting hemodynamics, could reconcile the diagnosis. In fact, the persistence of this communication could eventually perturb shear stress and favor a diagnosis of thrombosis. PDB is also associated with local atherosclerosis and calcification [19].

## Conclusions

Many cases similar to the one presented here are encountered during common clinical practice, in which the diagnosis cannot definitively be proven but is the most likely possibility. In the present case, the diagnosis was achieved via systematic use of imaging procedures and reconsideration of blood tests that explored thrombosis. This allowed a deeper and more detailed analysis of the case beyond the conventional approach that would have aimed to identify one cause for the condition at hand, in this case, atrial fibrillation. This broader approach resulted in the diagnosis of multiple embolisms from multiple sites and as a result of multiple causes.

## Abbreviations

JAK2: Janus kinase 2; PDB: patent ductus botalli.

## Consent

Written informed consent was obtained from the patient for publication of this case report and any accompanying images. A copy of the written consent is available for review by the Editor-in-Chief of this journal.

## Competing interests

The authors declare that they have no competing interests.

## Authors' contributions

LI interpreted the patient data regarding the multiple thrombosis and was a major contributor to the writing of the manuscript. MMis and AP collected the clinical data and reviewed the literature. MMis, AV, MMas, and GB were in charge of patient management for the surgical procedures. MR obtained and interpreted the echocardiographic studies. GC conducted JAK2 mutation analysis. All authors read and approved the final manuscript.
